# Ideal Cardiovascular Health Metrics Attenuated Association of Age at Menarche With Type 2 Diabetes in Rural China

**DOI:** 10.3389/ijph.2022.1604261

**Published:** 2022-08-30

**Authors:** Xueyan Wu, Lei Bao, Xiaotian Liu, Wei Liao, Ning Kang, Shengxiang Sang, Tanko Abdulai, Zhihan Zhai, Chongjian Wang, Yuqian Li

**Affiliations:** ^1^ Department of Epidemiology and Biostatistics, College of Public Health, Zhengzhou University, Zhengzhou, China; ^2^ The International Peace Maternity and Child Health Hospital, School of Medicine, Shanghai Jiao Tong University, Shanghai, China; ^3^ Department of Clinical Pharmacology, School of Pharmaceutical Science, Zhengzhou University, Zhengzhou, China

**Keywords:** rural population, age at menarche, ideal cardiovascular health metrics, type 2 diabetes, postmenopausal women

## Abstract

**Objective:** It is not clear whether ideal cardiovascular health (ICH) metrics have an impact on the association between age at menarche and type 2 diabetes (T2DM) in rural postmenopausal Chinese women.

**Methods:** In all, 15,450 postmenopausal women were enrolled from the Henan Rural Cohort study. Logistic regression models and interaction plots were used to analyze associations between age at menarche, ICH metrics and T2DM and interactive effects.

**Results:** Age at menarche was inversely associated with risk of T2DM, with adjusted OR of 1.224, 1.116, 1.00 and 0.971, 0.850 for those with age at menarche ≤13, 14, 15–16 (reference), 17, and ≥18 years, respectively, and each year of delay in menarche age correlated with a 5.1% lower risk of T2DM. Negative interaction effects of age at menarche and number of ICH metrics on the risk of T2DM was observed.

**Conclusion:** Meeting more ICH metrics might attenuate the association between early menstrual age and increased risk of T2DM, implying that meeting a higher number of ICH metrics may be an effective way to prevent T2DM for women of early menarche age.

## Introduction

Type 2 diabetes (T2DM) is expanding at an alarming rate worldwide, and its associated morbidity and mortality have caused a catastrophic socioeconomic cost [1]. The International Diabetes Federation reported that the prevalence of diabetes has reached global epidemic levels [2]. There are already 537 million people (20–79 years old) with diabetes, of which 140.9 million are Chinese [3]. Besides, with an aging population and rapidly changing lifestyles, diabetes has reached epidemic proportions in China during recent decades [4]. There is growing evidence that an earlier age at menarche was associated with an increased risk of obesity [5, 6] and T2DM [7–10]. However, there were inconsistent findings, with some studies finding no association between age at menarche and risk of developing diabetes [11].

Numerous studies have shown the impact of co-occurring lifestyle behaviors on diabetes, such as smoking and alcohol abuse, and physical activity [12, 13]. The American Heart Association (AHA) formulated the concept of ideal cardiovascular health (ICH) in 2010, which includes 4 health behavior metrics (smoking status, body mass index (BMI), physical activity, and diet) and 3 health factor metrics (total cholesterol (TC), blood pressure (BP), and fasting plasma glucose (FPG)) [14]. Previous studies have indicated an inverse association between the number of ICH metrics and the risks of T2DM [15, 16]. However, the impact of these independent and combined ideal cardiovascular health metrics on the relationship between age at menarche and the risk of T2DM is uncertain, especially in resource-limited populations, such as rural populations.

Hence, this study aimed to explore the impact of these independent and combined ideal cardiovascular health metrics on the relationship between age at menarche and the risk of T2DM in a Chinese rural population.

## Methods

### Study Design and Participants

Participants in this study were from the Henan Rural Cohort. The details of the Henan Rural Cohort have been described elsewhere [17]. Briefly, this rural-based study began in 2015 in Henan Province, China. A total of 39,259 people (23,769 for women) aged 18–79 years were recruited in the cohort study with a response rate of 93.7%. A total of 15,450 post-menopausal participants were included in this study. The study was approved by the “Zhengzhou University Life Science Ethics Committee” [Ethics approval code: [2015] MEC (S128)]. Informed consent was provided by all participants.

### Assessment of Menarche

Age at menarche is the age at the first menstrual period. This information was obtained through a standard questionnaire by face-to-face interviews by professionally trained workers. Participants were asked: “When was your first menstrual period?” The age range for first menstruation was 8–22 years [18]. Based on quintiles, age at menarche was categorized into five groups: ≤13, 14, 15 to16, 17, and ≥18 years. The early menarche group was defined as the onset of puberty at or before 13 years of age, and the third group (15–16 years) was the control group [19]. The last quintile (≥18 years) was defined as the late menarche menstruation group [19].

### Definition of Type 2 Diabetes

T2DM Patients were defined as those who met any of the following three criteria: 1) defects in insulin secretion or action of the participants were not caused by type 1 diabetes mellitus and gestational diabetes mellitus; 2) the patient had been diagnosed with T2DM by physicians and using anti-glycemic drugs in the last 2 weeks; and 3) the fasting blood glucose level exceeded 7.0 mmol/L [20].

### Ideal Cardiovascular Health Metrics

Each ICH metric was categorized as ideal and non-ideal according to the AHA definitions as the following [14]: ideal TC, TC < 5.18 mmol/L untreated; ideal FPG, FPG <5.6 mmol/L untreated; ideal BP, SBP <120/DBP< 80 mm Hg untreated; ideal smoking status, never a smoker; ideal physical activity, physical activity ≥150 min/wk of moderate intensity or ≥75 min/wk of vigorous intensity or ≥150 min/wk of moderate-vigorous intensity combination; ideal BMI, BMI <25 kg/m^2^; ideal diet, ≥ 4 components. A healthy diet score that had been appropriately adjusted was calculated by adding the number of diet components, including fruits and vegetables ≥500 g/d; fish ≥200 g/week; soybean products ≥125 g/d; red meat <75 g/d; drinking tea. Ideal diet was defined as healthy diet score ≥4 components [21]. The number of ICH metrics was calculated by summing up the number of ideal metrics for each participant. The more details of ICH have been described previously [22]. Since FPG is used to define T2DM, FPG was not used as a dimension of ICH in the current analysis [23].

### Assessment of Covariates

Information on demographic characteristics, lifestyles, behaviors, dietary patterns, individual history of diseases and medication use, and reproductive factors (use of oral contraceptive pills, parity, menopause status, age at menopause, and type of menopause) were collected using a standard questionnaire. The Food Frequency Questionnaire (FFQ) was based on the Dietary Guidelines for Chinese Residents and their eating habits. Previous studies have shown that the FFQ has good reproducibility and validity [24]. Weight and height were measured twice in light clothing with shoes off and recorded to the nearest 0.1kg and 0.1 cm respectively and we calculated the average of the two measures. Body mass index (BMI) was computed as body weight (kg) divided by height square (m^2^) based on the measurement. Blood pressure was measured three times by electronic sphygmomanometer (Omron HEM-7071A, Japan) in the right arm in a sitting position after at least 5 min rest. There were 30s intervals between the three measurements. Venous blood samples were collected from subjects after an overnight fast of at least 8 h and stored in a −80°C cryogenic refrigerator before analysis. The fasting blood glucose (FBG) was analyzed via glucose oxidative method (GOD-PAP) by ROCHE Cobas C501 automatic biochemical analyzer. Total cholesterol was measured by Roche Cobas C501 automatic biochemical analyzer. The details of the equipment for anthropometric and clinical examinations have been introduced elsewhere [17].

### Statistical Analysis

Continuous and categorical variables were presented as mean ± standard deviation (SD) and number (percentage), respectively. The participants’ baseline characteristics were compared using one-way analysis of variance and Pearson’s χ^2^ test. Ten models were developed to assess associations of age at menopause, ICH metrics and the risk of T2DM by using logistic regression models: Model 1 adjusted for age; Model 2 adjusted as in model 1 plus education level, average monthly individual income, marital status, alcohol drinking; Model 3 adjusted as in Model 2 plus family history of diabetes, age at menopause, parity, the cause of the menopause, use of oral contraceptive pills; Model 4 adjusted as in Model 3 plus physical activity; Model 5 adjusted as in Model 3 plus body mass index; Model 6 adjusted as in Model 3 plus smoking; Model 7 adjusted as in Model 3 plus diet; Model 8 adjusted as in Model 3 plus blood pressure; Model 9 adjusted as in Model 3 plus total cholesterol; Model 10 adjusted as in Model 3 plus physical activity, body mass index, smoking, diet, blood pressure, total cholesterol. Additionally, tests for linear trends were conducted by modeling the five categories of menarche age (≤13, 14, 15 to16, 17, and ≥18 years) as a continuous variable in the regression model. The interaction effects of age at menopause and ICH metrics on the risk of T2DM by using the generalized linear regression model and visualized by using interplot method. All statistical analyses were performed by STATA 15 for Windows and R version 3.6.3, and the statistical significance was set *p*-value < 0.05 at two tails.

## Results

### Participants Characteristics

Characteristics of the participants are provided in Table 1. Among 15,450 postmenopausal women, 1959 cases of T2DM were identified. The mean ± SD age of the menopausal women participants, menarche, and menopause were 61.5 ± 7.71 years, 16.17 ± 2.26, and 48.78 ± 4.71, respectively. Women with T2DM tended to have a lower education level and lower average monthly income compared to those without T2DM. Compared to women with an age at menarche of 15–16 years (reference group), those who had early menarche (≤13 years) were much younger, had a higher education level, higher monthly individual income, and higher FPG, and had a higher prevalence of T2DM (Supplementary Table S1).

**TABLE 1:** Distributions of selected variables of the study participant stratified according to type 2 diabetes mellitus status (The Henan Rural Cohort Study, collected during 2015–2017, China).

Variables	Total	Non-T2DM	T2DM	P
N (%)	15,450 (100)	13,491 (87.32)	1959 (12.68)	<0.001
Age (year, mean ± SD)	61.50 ± 7.71	61.26 ± 7.79	63.14 ± 6.97	<0.001
Education level (n, %)				<0.001
Elementary school or below	9,947 (64.38)	8,523 (63.18)	1,424 (72.69)	
Middle school	4,281 (27.71)	3,852 (28.55)	429 (21.90)	
High school or above	1,222 (7.91)	1,116 (8.27)	106 (5.41)	
Average monthly income (n, %)				0.012
<500	6,278 (40.63)	5,438 (40.31)	840 (42.88)	
500∼	4,964 (32.13)	4,327 (32.07)	637 (32.52)	
1,000∼	4,208 (27.24)	3,726 (27.62)	482 (24.60)	
Current regular smoker (n, %)	50 (0.32)	42 (0.31)	8 (0.41)	0.702
Current regular drinking (n, %)	317 (2.05)	292 (2.16)	25 (1.28)	0.023
Family history of diabetes (n, %)	500 (3.24)	330 (2.45)	170 (8.68)	<0.001
Age at menarche (year, mean ± SD)	16.17 ± 2.26	16.18 ± 2.25	16.12 ± 2.33	0.252
Age at menopause (year, mean ± SD)	48.78 ± 4.71	48.76 ± 4.67	48.91 ± 4.98	0.212
Natural menopause (Yes, n(%))	14,072 (91.08)	12,262 (90.89)	1810 (92.39)	0.074
Use of oral contraceptive pills (Yes, n(%))	261 (1.69)	240 (1.78)	21 (1.08)	0.024
Parity (n, %)				<0.001
0∼	72 (0.47)	62 (0.46)	10 (0.51)	
1∼	7,693 (49.82)	6,844 (50.76)	849 (43.34)	
3∼	7,677 (49.72)	6,577 (48.78)	1,100 (56.15)	
FPG (mmol/L, mean ± SD)	5.72 ± 1.63	5.28 ± 0.59	8.77 ± 2.83	<0.001

T2DM, type 2 diabetes mellitus; SD, standard deviation; Average monthly income, Renminbi; FPG, fasting plasma glucose.

### Association of Age at Menarche, Ideal Cardiovascular Health Metrics, and the Risk of Type 2 Diabetes

The distributions of the ideal cardiovascular health metrics according age at menarche is presented in Supplementary Table S2. There were significant differences in the number of ICH metrics among the different age at menarche groups. Women with a younger age at menarche were more likely to not have an ideal BMI, ideal physical activity, and ideal BP (all P _trend_ <0.05), and tended to have fewer numbers of ICH metrics. After adjustment for confounding factors, age at menarche was inversely associated with risk of T2DM, with adjusted OR (95% CI) of 1.224 (1.043, 1.436), 1.116(0.945, 1.318), 1.00, 0.971 (0.834, 1.130), and 0.850 (0.749, 0.965) for those with age at menarche ≤13, 14, 15–16 (reference), 17, and ≥18 years, respectively. Each year of delay in menarche age correlated with a 5.1% lower risk of T2DM after adjustment for potential confounding factors (OR (95% CI) = 0.949 (0.928, 0.971)) (Table 2). From Model 4 to Model 10, this association changed slightly after adjusting for individual ICH metrics but remained statistically significant. The model fits were shown in Supplementary Table S3. In models 4, 5, 9 and 10, the Hosmer and Lemeshow goodness of fit test is not statistically significant, which indicates that the model fits well. To assess the robustness of the association between age at menarche and the development of T2DM, women with co-morbidities such as cancer and kidney failure were further excluded for sensitivity analysis (Supplementary Table S4), and the strength of the association in the sensitivity analysis was very similar to that found in Table 2. However, in Model 5 (further adjustment for BMI), no association was observed between age at menarche and T2DM.

**TABLE 2:** Association of age at menarche (years) with type 2 diabetes mellitus in rural Chinese women (The Henan Rural Cohort Study, collected during 2015–2017, China).

Models	≤13	14	15∼16	17	≥18	Per 1-year increase
Model 1	1.226 (1.048, 1.434)	1.098 (0.933, 1.292)	Ref.	0.974 (0.838, 1.131)	0.858 (0.756, 0.972)	0.951 (0.930, 0.973)
Model 2	1.247 (1.065, 1.458)	1.112 (0.945, 1.309)	Ref.	0.964 (0.829, 1.120)	0.845 (0.745, 0.957)	0.947 (0.926, 0.968)
Model 3	1.224 (1.043, 1.436)	1.116 (0.945, 1.318)	Ref.	0.971 (0.834, 1.130)	0.850 (0.749, 0.965)	0.949 (0.928, 0.971)
Model 4	1.230 (1.048, 1.444)	1.114 (0.943, 1.316)	Ref.	0.976 (0.838, 1.136)	0.854 (0.752, 0.970)	0.950 (0.929, 0.972)
Model 5	1.200 (1.022, 1.409)	1.108 (0.938, 1.309)	Ref.	0.993 (0.853, 1.157)	0.894 (0.787, 1.016)	0.960 (0.939, 0.982)
Model 6	1.224 (1.043, 1.436)	1.116 (0.945, 1.317)	Ref.	0.971 (0.834, 1.131)	0.850 (0.748, 0.965)	0.949 (0.928, 0.971)
Model 7	1.242 (1.058, 1.459)	1.130 (0.956, 1.335)	Ref.	1.004 (0.862, 1.170)	0.883 (0.777, 1.003)	0.955 (0.933, 0.977)
Model 8	1.244 (1.059, 1.461)	1.139 (0.964, 1.345)	Ref.	0.975 (0.837, 1.136)	0.865 (0.761, 0.982)	0.950 (0.928, 0.971)
Model 9	1.224 (1.043, 1.436)	1.116 (0.945, 1.318)	Ref.	0.971 (0.834, 1.130)	0.850 (0.749, 0.966)	0.949 (0.928, 0.971)
Model 10	1.241 (1.055, 1.459)	1.139 (0.963, 1.348)	Ref.	1.023 (0.877, 1.194)	0.927 (0.815, 1.054)	0.963 (0.941, 0.985)

Data are odds ratios (95% confidence intervals).

Model 1: adjusted for age; Model 2: adjusted as in model 1 plus education level, average monthly individual income, marital status, alcohol drinking; Model 3: adjusted as in model 2 plus family history of diabetes, Age at menopause, parity, the cause of the menopause, use of oral contraceptive pills; Model 4: adjusted as in model 3 plus physical activity; Model 5: adjusted as in model 3 plus body mass index; Model 6: adjusted as in model 3 plus smoking; Model 7: adjusted as in model 3 plus diet; Model 8: adjusted as in model 3 plus blood pressure; Model 9: adjusted as in model 3 plus total cholesterol; Model 10: adjusted as in model 3 plus physical activity, body mass index, smoking, diet, blood pressure, total cholesterol.

### Interactive Effect of Age at Menarche and Ideal Cardiovascular Health Metrics on the Risk of Type 2 Diabetes

**FIGURE 1:**
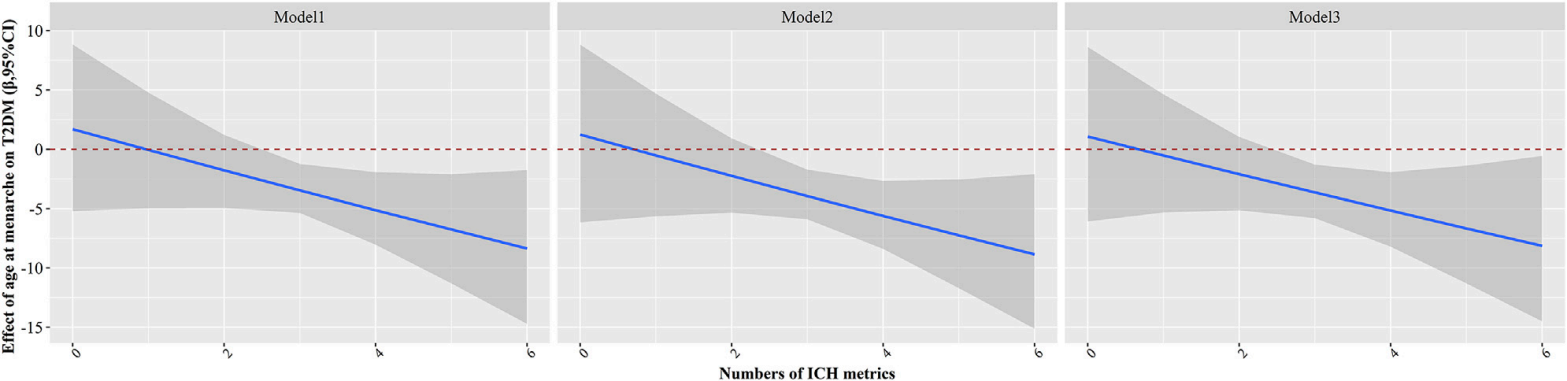
Estimated effects of age at menarche on type 2 diabetes mellitus as a function of number of ideal cardiovascular health metrics were analyzed by using generalized linear models (The Henan Rural Cohort Study, collected during 2015–2017, China). The black lines and grey areas represented the estimated effect and 95% confidence interval of age at menarche on type 2 diabetes mellitus along with altered values of number of ideal cardiovascular health metrics. Model 1: adjusted for age; Model 2: adjusted as in model 1 plus education level, average monthly individual income, marital status, alcohol drinking; Model 3: adjusted as in model 2 plus family history of diabetes, Age at menopause, parity, the cause of the menopause, use of oral contraceptive pills.

The stratified associations between age at menarche and the risk of T2DM in each ICH metrics are presented in Table 3. There was a significant interaction between age at menarche and physical activity, BMI, TC, and BP levels in the development of T2DM (all P for interaction <0.001). The risk of T2DM was more predominant in women with age at menarche ≤13 years and non-ideal BMI (OR (95% CI), 1.263 (1.031, 1.548)) than in women with ideal BMI (OR (95% CI), 1.230 (0.940, 1.610)) (*p* < 0.001 for interaction). The estimated values were similarly higher in the group with non-ideal total cholesterol levels (OR (95% CI), 1.247 (1.000, 1.556)) than in the group with ideal total cholesterol levels (OR (95% CI), 1.226 (0.965, 1.557)) (*p* < 0.001 for interaction) and in the group with non-ideal blood pressure levels (OR (95% CI), 1.229 (1.025, 1.474)) than in the ideal blood pressure level group (OR (95% CI), 1.280 (0.890, 1.840)) (*p* < 0.001 for interaction). The combined effect of the number of ICH metrics and age at menarche on the risk of T2DM is illustrated in Table 3 and Figure 1. We found a significant interaction between individual participants meeting the number of ICH metrics and age at menarche on the risk of T2DM. Figure 1 shows that the estimated effect of age at menarche on the risk of T2DM changes with the increasing number of ICH metrics. Negative interaction effects of age at menarche and number of ICH metrics on the risk of T2DM was observed in all participants (*p* < 0.05). Furthermore, the associations of age at menarche with the risk of T2DM were completed counteracted by the number of ICH metrics at the cut-points of 3.

**TABLE 3:** The association between age at menarche (years) and type 2 diabetes mellitus according to ideal cardiovascular health metrics (The Henan Rural Cohort Study, collected during 2015–2017, China).

Variables	≤13	14	15∼16	17	≥18	*p* for interaction
Diet						0.993
Ideal	NA	NA	Ref.	NA	NA	
Non-ideal	1.238 (1.053, 1.456)	1.134 (0.958, 1.341)	Ref.	1.022 (0.876, 1.193)	0.925 (0.813, 1.052)	
Physical activity						<0.001
Ideal	1.262 (1.068, 1.491)	1.172 (0.985, 1.393)	Ref.	1.003 (0.854, 1.178)	0.911 (0.796, 1.043)	
Non-ideal	1.029 (0.504, 2.102)	0.795 (0.395, 1.600)	Ref.	1.317 (0.757, 2.290)	1.114 (0.714, 1.740)	
Smoking						0.130
Ideal	1.230 (1.046, 1.447)	1.125 (0.950, 1.331)	Ref.	1.012 (0.868, 1.181)	0.924 (0.812, 1.051)	
Non-ideal	NA	NA	Ref.	NA	NA	
BMI						<0.001
Ideal	1.230 (0.940, 1.610)	1.284 (0.987, 1.670)	Ref.	0.862 (0.674, 1.103)	0.894 (0.737, 1.085)	
Non-ideal	1.263 (1.031, 1.548)	1.063 (0.855, 1.323)	Ref.	1.154 (0.946, 1.407)	0.961 (0.808, 1.143)	
Total cholesterol						<0.001
Ideal	1.226 (0.965, 1.557)	1.163 (0.911, 1.486)	Ref.	0.980 (0.773, 1.242)	0.948 (0.780, 1.151)	
Non-ideal	1.247 (1.000, 1.556)	1.114 (0.884, 1.405)	Ref.	1.058 (0.863, 1.296)	0.906 (0.762, 1.077)	
Blood pressure						<0.001
Ideal	1.280 (0.890, 1.840)	1.104 (0.751, 1.622)	Ref.	0.896 (0.620, 1.293)	0.686 (0.495, 0.951)	
Non-ideal	1.229 (1.025, 1.474)	1.148 (0.952, 1.384)	Ref.	1.053 (0.888, 1.248)	0.983 (0.854, 1.131)	
Number of ICH metrics						<0.001
0∼	1.279 (0.967, 1.690)	1.026 (0.759, 1.387)	Ref.	1.225 (0.944, 1.590)	0.980 (0.782, 1.228)	
3∼	1.232 (1.002, 1.514)	1.133 (0.916, 1.400)	Ref.	0.916 (0.751, 1.119)	0.895 (0.761, 1.052)	
5∼	0.922 (0.447, 1.900)	1.578 (0.840, 2.967)	Ref.	0.822 (0.424, 1.594)	0.664 (0.377, 1.169)	

Note: Results were adjusted for age, education level, average monthly individual income, marital status, alcohol drinking, family history of diabetes, Age at menopause, parity, the cause of the menopause, use of oral contraceptive pills. Participants’ cardiovascular health metrics were mutually adjusted.

NA, not applicable; BMI, body mass index; BP, blood pressure; TC, total cholesterol; FPG, fasting plasma glucose; ICH, ideal cardiovascular health.

## Discussion

To the best of our knowledge, this is the first study to explore the impact of ICH on the association between age at menarche and risk of T2DM in rural postmenopausal Chinese women, which provided important new evidence in a Chinese rural population. Our study further suggests that the early age at menarche was associated with increased risk of T2DM. The more significant finding is that ICH had a protective effect against the negative effect of age at menarche on the risk of T2DM, and the associations of age at menarche with the risk of T2DM were completely counteracted by the number of ICH metrics at the cut-points of 3.

Our results are generally consistent with other studies. A finding from the REACTION study indicated that age at menarche was inversely associated with the risk of diabetes in adulthood in Chinese women [25]. The EPIC-InterAct study found that women with a history of early menarche have a higher risk of type 2 diabetes in adulthood [26]. Similar results were found in our study. Moreover, numerous studies showed that age at menarche is associated with various forms of dysglycemia [6, 7, 27]. However, some studies have shown inconsistent results. The Rancho Bernardo Study showed that age at menarche was not associated with AGT or type 2 diabetes risk [28]. A study for postmenopausal women from Fujian Province indicated that age at menarche was not associated with diabetes [11]. Atherosclerosis Risk in Communities (ARIC) study found that early menarche was associated with type 2 diabetes in White women, and a similar association was not found in African-American women [29]. The difference between our findings and those of other studies may be due to the different populations, race/ethnic differences, and different nutrition and lifestyles among the participants [19, 29].

Our further study found that ICH had a protective effect against the negative effect of age at menarche on the risk of T2DM. Previous studies have indicated the inverse association between the number of ICH metrics and the risks of type 2 diabetes mellitus [15, 16]. Moreover, the Jackson Heart Study discovered that the AHA concept of ICH is applicable to diabetes mellitus prevention among Black participants [30]. A follow-up study found that participants with achieved ≥2 of ICH metrics had a lower OR of T2DM compared to those who achieved ≥2 of ICH metrics [31]. One recent study found that the increased risk of diabetes related to earlier age at menarche was significantly attenuated among individuals with ≥4 ICH metrics [25]. Similar results were found in our study, showing that the association between age at menarche and risk of T2DM was counteracted when the number of ICH metrics was ≥3. This evidence suggests that the beneficial effects on human health of achieving a higher number of ICH metrics may outweigh the adverse health effects of an early age of menarche. In addition, we found that separate ICH metrics, including physical activity, BMI, TC, and BP interacted meaningfully with age at menarche in the risk of T2DM. The risk of T2DM was more predominant in women with age at menarche ≤13 years and a non-ideal BMI than in women with an ideal BMI. The estimated values were similarly higher in the group with non-ideal TC and BP. Our previous study found that the risk of T2DM was higher among women who had an early menarche, and the relationship was partially mediated by BMI, and the proportion of the effect was 28% [19], which reflected the importance of meeting the ideal BMI for health. Abnormal cholesterol metabolism played an important role in diabetes [32, 33]. Some studies have indicated that early age at menarche was positively associated with hypertension [34, 35]. Control of BP, lipid, and glycemia were essential components for the prevention of type 2 diabetes complications [36]. Our study further supports that an increased number of ICH metrics is beneficial in reducing the risk of T2DM for women with early age at menarche. However, women who were ≤13 years of age at menarche with ideal physical activity or ideal smoking status were more likely to be at risk for T2DM. This could be because the prevalence of smoking among women is much lower in China and the population selected for this study was menopausal women. Additionally, our preliminary study found that the ideal physical activity (91.37%) was the highest prevalence of ICH metrics in Chinese rural population.

### Strengths and Limitations

Our study is based on the relatively large sample size of a rural population in China. Standardized investigation tools, training, and on-site implementation, as well as adjustments of a wide range of potential confounding factors, ensure the reliability of the analysis. Furthermore, this is the first study to explore the impact of ICH on the association between age at menarche and risk of T2DM among rural postmenopausal Chinese women. Nevertheless, several limitations should also be considered. First, these findings come from a cross-sectional study, rather than a prospective cohort design, thus do not accurately describe causality. Secondly, our study did not use an oral glucose tolerance test to define diabetes, which may underestimate the number of diabetics, and the menarche age was obtained by self-reporting, leading to possible recall bias. However, there were high correlations between the recalled and actual menarche age [37, 38], reporting error likely would be irrelevant with T2DM status.

### Conclusion

The results indicated that early age at menarche was related to increased T2DM risk and those associations were attenuated by higher ICH metrics, indicating that ICH may be an effective method to reduce the burden of T2DM among rural Chinese women with early menarche age.

## References

[1] World Health Organization. Global Report on Diabetes. Working Papers (2016).

[2] Zareban I Karimy M Niknami S Haidarnia A Rakhshani F. The Effect of Self-Care Education Program on Reducing HbA1c Levels in Patients with Type 2 Diabetes. J Edu Health Promot (2014) 3:123. 10.4103/2277-9531.145935 PMC4275624 25540796

[3] Federation ID Diabetes Around the World in 2021 (2021). Available at: https://diabetesatlas.org/ (Accessed December 9, 2021).

[4] Xu Y Wang L He J Bi Y Li M Wang T Prevalence and Control of Diabetes in Chinese Adults. JAMA (2013) 310(9):948–59. 10.1001/jama.2013.168118 24002281

[5] Lakshman R Forouhi N Luben R Bingham S Khaw K Wareham N Association between Age at Menarche and Risk of Diabetes in Adults: Results from the EPIC-Norfolk Cohort Study. Diabetologia (2008) 51(5):781–6. 10.1007/s00125-008-0948-5 18320165

[6] Sumi A Iwase M Nakamura U Fujii H Ohkuma T Ide H Impact of Age at Menarche on Obesity and Glycemic Control in Japanese Patients with Type 2 Diabetes: Fukuoka Diabetes Registry. J Diabetes Investig (2018) 9(5):1216–23. 10.1111/jdi.12839 PMC6123051 29575815

[7] Stöckl D Döring A Peters A Thorand B Heier M Huth C Age at Menarche is Associated with Prediabetes and Diabetes in Women (Aged 32-81 Years) from the General Population: the KORA F4 Study. Diabetologia (2012) 55(3):681–8. 10.1007/s00125-011-2410-3 22170465

[8] Lim JS Lee HS Kim EY Yi KH Hwang JS. Early Menarche Increases the Risk of Type 2 Diabetes in Young and Middle-Aged Korean Women. Diabet Med (2015) 32(4):521–5. 10.1111/dme.12653 25441051

[9] Pandeya N Huxley RR Chung H-F Dobson AJ Kuh D Hardy R Female Reproductive History and Risk of Type 2 Diabetes: A Prospective Analysis of 126 721 Women. Diabetes Obes Metab (2018) 20(9):2103–12. 10.1111/dom.13336 29696756 PMC6105508

[10] Yang L Li L Peters SAE Clarke R Guo Y Chen Y Age at Menarche and Incidence of Diabetes: A Prospective Study of 300,000 Women in China. Am J Epidemiol (2018) 187(2):190–8. 10.1093/aje/kwx219 28605451 PMC5860078

[11] Qiu C Chen H Wen J Zhu P Lin F Huang B Associations between Age at Menarche and Menopause with Cardiovascular Disease, Diabetes, and Osteoporosis in Chinese Women. J Clin Endocrinol Metab (2013) 98(4):1612–21. 10.1210/jc.2012-2919 23471979

[12] Long GH Johansson I Rolandsson O Wennberg P Fhärm E Weinehall L Healthy Behaviours and 10-year Incidence of Diabetes: A Population Cohort Study. Prev Med (2015) 71:121–7. 10.1016/j.ypmed.2014.12.013 25532678

[13] Shan Z Li Y Zong G Guo Y Li J Manson JE Rotating Night Shift Work and Adherence to Unhealthy Lifestyle in Predicting Risk of Type 2 Diabetes: Results from Two Large US Cohorts of Female Nurses. BMJ (2018) 363:k4641. 10.1136/bmj.k4641 30464025 PMC6247172

[14] Lloyd-Jones DM Hong Y Labarthe D Mozaffarian D Appel LJ Van Horn L Defining and Setting National Goals for Cardiovascular Health Promotion and Disease Reduction: the American Heart Association's Strategic Impact Goal through 2020 and beyond. Circulation (2010) 121(4):586–613. 10.1161/circulationaha.109.192703 20089546

[15] Joseph JJ Bennett A Echouffo Tcheugui JB Effoe VS Odei JB Hidalgo B Ideal Cardiovascular Health, Glycaemic Status and Incident Type 2 Diabetes Mellitus: the REasons for Geographic and Racial Differences in Stroke (REGARDS) Study. Diabetologia (2019) 62(3):426–37. 10.1007/s00125-018-4792-y 30643923 PMC6392040

[16] Joseph JJ Echouffo-Tcheugui JB Carnethon MR Bertoni AG Shay CM Ahmed HM The Association of Ideal Cardiovascular Health with Incident Type 2 Diabetes Mellitus: The Multi-Ethnic Study of Atherosclerosis. Diabetologia (2016) 59(9):1893–903. 10.1007/s00125-016-4003-7 27272340 PMC4970884

[17] Liu X Mao Z Li Y Wu W Zhang X Huo W Cohort Profile: The Henan Rural Cohort: A Prospective Study of Chronic Non-communicable Diseases. Int J Epidemiol (2019) 48:1756–j. 10.1093/ije/dyz039 30915440

[18] Liu G Yang Y Huang W Zhang N Zhang F Li G Association of Age at Menarche with Obesity and Hypertension Among Southwestern Chinese Women: A New Finding. Menopause (2018) 25(5):546–53. 10.1097/GME.0000000000001027 29112597

[19] Zhang L Li Y Wang C Mao Z Zhou W Tian Z Early Menarche is Associated with an Increased Risk of Type 2 Diabetes in Rural Chinese Women and is Partially Mediated by BMI: The Henan Rural Cohort Study. Menopause (New York, N.Y.) (2019) 26(11):1265–71. 10.1097/GME.0000000000001385 31688573

[20] American Diabetes Association. 2. Classification and Diagnosis of Diabetes: Standards of Medical Care in Diabetes-2020. Diabetes Care (2020) 43(Suppl. 1):S14–S31. 10.2337/dc20-S002 31862745

[21] Han C Liu F Yang X Chen J Li J Cao J Ideal Cardiovascular Health and Incidence of Atherosclerotic Cardiovascular Disease Among Chinese Adults: The China-PAR Project. Sci China Life Sci (2018) 61(5):504–14. 10.1007/s11427-018-9281-6 29721777

[22] Wu X Liu X Liao W Kang N Sang S Abdulai T Association of Night Sleep Duration and Ideal Cardiovascular Health in Rural China: The Henan Rural Cohort Study. Front Public Health (2021) 8:606458. 10.3389/fpubh.2020.606458 33505951 PMC7830879

[23] Climie RE van Sloten TT Périer M-C Tafflet M Fayosse A Dugravot A Change in Cardiovascular Health and Incident Type 2 Diabetes and Impaired Fasting Glucose: The Whitehall II Study. Diabetes Care (2019) 42(10):1981–7. 10.2337/dc19-0379 31416895 PMC7364667

[24] Xue Y Yang K Wang B Liu C Mao Z Yu S Reproducibility and Validity of an FFQ in the Henan Rural Cohort Study. Public Health Nutr (2019) 23:34–40. 10.1017/s1368980019002416 31474239 PMC10200648

[25] Hu C Zhang Y Zhang J Huo Y Wan Q Li M Age at Menarche, Ideal Cardiovascular Health Metrics, and Risk of Diabetes in Adulthood: Findings from the REACTION Study. J Diabetes (2020) 13:458–68. 10.1111/1753-0407.13128 33135296

[26] Elks CE Ong KK Scott RA van der Schouw YT Brand JS Wark PA Age at Menarche and Type 2 Diabetes Risk. Diabetes Care (2013) 36(11):3526–34. 10.2337/dc13-0446 24159179 PMC3816901

[27] Baek T-H Lim N-K Kim M-J Lee J Ryu S Chang Y Age at Menarche and its Association with Dysglycemia in Korean Middle-Aged Women. Menopause (2015) 22(5):542–8. 10.1097/GME.0000000000000353 25335102 PMC4470522

[28] Saquib N Kritz-Silverstein D Barrett-Connor E. Age at Menarche, Abnormal Glucose Tolerance and Type 2 Diabetes Mellitus: The Rancho Bernardo Study. Climacteric (2005) 8(1):76–82. 10.1080/13697130500062688 15804735

[29] Dreyfus JG Lutsey PL Huxley R Pankow JS Selvin E Fernández-Rhodes L Age at Menarche and Risk of Type 2 Diabetes Among African-American and white Women in the Atherosclerosis Risk in Communities (ARIC) Study. Diabetologia (2012) 55(9):2371–80. 10.1007/s00125-012-2616-z 22760786 PMC3690318

[30] Effoe VS Carnethon MR Echouffo‐Tcheugui JB Chen H Joseph JJ Norwood AF The American Heart Association Ideal Cardiovascular Health and Incident Type 2 Diabetes Mellitus Among Blacks: The Jackson Heart Study. J Am Heart Assoc (2017) 6(6):e005008. 10.1161/JAHA.116.005008 28637777 PMC5669153

[31] Fretts AM Howard BV McKnight B Duncan GE Beresford SAA Mete M Life's Simple 7 and Incidence of Diabetes Among American Indians: The Strong Heart Family Study. Diabetes Care (2014) 37(8):2240–5. 10.2337/dc13-2267 24804696 PMC4113167

[32] Wilund K Feeney L Tomayko E Weiss E Hagberg J. Effects of Endurance Exercise Training on Markers of Cholesterol Absorption and Synthesis. Physiol Res (2009) 58(4):545–52. 10.33549/physiolres.931515 18656998

[33] Mokdad AH Bowman BA Ford ES Vinicor F Marks JS Koplan JP. The Continuing Epidemics of Obesity and Diabetes in the United States. JAMA (2001) 286(10):1195–200. 10.1001/jama.286.10.1195 11559264

[34] Bubach S De Mola CL Hardy R Dreyfus J Santos AC Horta BL. Early Menarche and Blood Pressure in Adulthood: Systematic Review and Meta-Analysis. J Public Health (2018) 40(3):476–84. 10.1093/pubmed/fdx118 28977577

[35] Zhang L Li Y Zhou W Wang C Dong X Mao Z Mediation Effect of BMI on the Relationship between Age at Menarche and Hypertension: The Henan Rural Cohort Study. J Hum Hypertens (2020) 34(6):448–56. 10.1038/s41371-019-0247-2 31477825

[36] Janghorbani M Papi B Amini M. Current Status of Glucose, Blood Pressure and Lipid Management in Type 2 Diabetes Clinic Attendees in Isfahan, Iran. J Diabetes Invest (2015) 6(6):716–25. 10.1111/jdi.12349 PMC4627550 26543547

[37] Must A Phillips SM Naumova EN Blum M Harris S Dawson-Hughes B Recall of Early Menstrual History and Menarcheal Body Size: After 30 Years, How Well Do Women Remember? Am J Epidemiol (2002) 155(7):672–9. 10.1093/aje/155.7.672 11914195

[38] Cooper R Blell M Hardy R Black S Pollard TM Wadsworth MEJ Validity of Age at Menarche Self-Reported in Adulthood. J Epidemiol Community Health (2006) 60(11):993–7. 10.1136/jech.2005.043182 17053289 PMC2465480

