# Supplementary material for: Ideal Cardiovascular Health Metrics Attenuated Association of Age at Menarche With Type 2 Diabetes in Rural China
Source: Int J Public Health. 2022 Aug 30;67:1604261. doi: 10.3389/ijph.2022.1604261 (PMC9469086; doi:10.3389/ijph.2022.1604261)
Supplement: Supplementary file 1 [file Table1.docx]

**Supplementary Table 1. Distributions of selected variables of the study participant stratified by age at menarche (years) (The Henan Rural Cohort Study, collected during 2015–2017, China)**

| **Variables** |  | **≤13** | **14** | **15~16** | **17** | **≥18** |  | ***P*_trend_** |
| --- | --- | --- | --- | --- | --- | --- | --- | --- |
| **N (%)** |  | 1875(12.14) | 1783(11.55) | 5124(33.18) | 2238(14.49) | 4422(28.64) |  |  |
| **Age (year, mean ± SD)** |  | 58.23±7.52 | 58.75±7.77 | 60.46±7.47 | 62.27±7.13 | 64.8±7.01 |  | <0.001 |
| **Education level (n, %)** |  |  |  |  |  |  |  | <0.001 |
| Elementary school or below |  | 972(51.81) | 941(52.75) | 3087(60.23) | 1513(67.51) | 3434(77.62) |  |  |
| Middle school |  | 661(35.23) | 639(35.82) | 1585(30.93) | 585(26.10) | 811(18.33) |  |  |
| High school or above |  | 243(12.95) | 204(11.43) | 453(8.84) | 143(6.38) | 179(4.05) |  |  |
| **Average monthly income (n, %)** |  |  |  |  |  |  |  | <0.001 |
| <500 |  | 692(36.89) | 638(35.76) | 1940(37.85) | 917(40.92) | 2091(47.26) |  |  |
| 500~ |  | 592(31.56) | 627(35.15) | 1745(34.05) | 726(32.40) | 1274(28.80) |  |  |
| 1000 ~ |  | 592(31.56) | 519(29.09) | 1440(28.10) | 598(26.68) | 1059(23.94) |  |  |
| **Current regular smoker (n, %)** |  | 4(0.21) | 7(0.39) | 14(0.27) | 3(0.13) | 22(0.50) |  | 0.265 |
| **Current regular drinking (n, %)** |  | 57(3.04) | 40(2.24) | 104(2.03) | 37(1.65) | 79(1.79) |  | 0.008 |
| **Family history of diabetes (n, %)** |  | 100(5.33) | 71(3.98) | 179(3.49) | 66(2.95) | 84(1.90) |  | <0.001 |
| **Age at menopause (year, mean ± SD)** | | 48.02±4.99 | 48.38±4.68 | 48.81±4.63 | 49.14±4.39 | 49.05±4.80 |  | <0.001 |
| **Natural menopause（Yes, n(%)）** |  | 1631(86.94) | 1567(87.84) | 4618(90.11) | 2086(93.08) | 4170(94.26) |  | <0.001 |
| **Use of oral contraceptive pills (n, %)** | | 43(2.30) | 28(1.58) | 81(1.58) | 45(2.01) | 64(1.45) |  | 0.140 |
| **Parity (n, %)** |  |  |  |  |  |  |  | <0.001 |
| 0~ |  | 11(0.59) | 13(0.73) | 20(0.39) | 11(0.49) | 17(0.38) |  |  |
| 1~ |  | 1143(60.96) | 1058(59.34) | 2742(53.51) | 1069(47.77) | 1681(38.01) |  |  |
| 3~ |  | 721(38.45) | 712(39.93) | 2362(46.10) | 1158(51.74) | 2724(61.60) |  |  |
| **FPG (mmol/L, mean ± SD)** |  | 5.76±1.77 | 5.70±1.60 | 5.73±1.61 | 5.74±1.68 | 5.7±1.57 | | 0.619 |
| **Prevalence of T2DM (95%CI)** |  | 13.81(12.24, 15.37) | 12.78(11.23, 14.33) | 12.41(11.51, 13.31) | 12.81(11.42, 14.19) | 12.41(11.44, 13.38) | | 0.283 |

SD, standard deviation; Average monthly income, Renminbi; FPG, fasting plasma glucose; T2DM, type 2 diabetes mellitus.

**Supplementary Table 2. Distributions of the ideal cardiovascular health metrics according age at menarche (years) (The Henan Rural Cohort Study, collected during 2015–2017, China)**

| **Variables (n, %)** |  | **≤13** | **14** | **15~16** | **17** | **≥18** |  | ***P*_trend_** |
| --- | --- | --- | --- | --- | --- | --- | --- | --- |
| **Ideal smoking status** |  | 1868(12.14) | 1776(11.55) | 5106(33.19) | 2235(14.53) | 4397(28.59) |  | 0.266 |
| **Ideal BMI** |  | 802(10.17) | 814(10.32) | 2466(31.27) | 1198(15.19) | 2607(33.05) |  | <0.001 |
| **Ideal physical activity** |  | 1814(12.34) | 1710(11.63) | 4875(33.16) | 2139(14.55) | 4162(28.31) |  | <0.001 |
| **Ideal diet** |  | 4(13.79) | 5(17.24) | 11(37.93) | 4(13.79) | 5(17.24) |  | 0.165 |
| **Ideal BP** |  | 604(13.62) | 556(12.54) | 1399(31.54) | 668(15.06) | 1208(27.24) |  | 0.001 |
| **Ideal TC** |  | 1068(12.60) | 1028(12.12) | 2751(32.44) | 1206(14.22) | 2426(28.61) |  | 0.076 |
| **Ideal FPG** |  | 1206(12.21) | 1137(11.51) | 3228(32.68) | 1457(14.75) | 2850(28.85) |  | 0.397 |
| **Number of ICH metrics** |  |  |  |  |  |  |  | <0.001 |
| ≤1 |  | 9(0.48) | 13(0.73) | 49(0.96) | 25(1.12) | 38(0.86) |  |  |
| 2 |  | 219(11.67) | 185(10.37) | 604(11.79) | 242(10.80) | 449(10.15) |  |  |
| 3 |  | 472(25.16) | 477(26.74) | 1327(25.89) | 523(23.34) | 1064(24.05) |  |  |
| 4 |  | 572(30.49) | 505(28.31) | 1581(30.85) | 666(29.72) | 1302(29.43) |  |  |
| 5 |  | 409(21.80) | 432(24.22) | 1111(21.68) | 545(24.32) | 1107(25.02) |  |  |
| ≥6 |  | 195(10.39) | 172(9.64) | 453(8.84) | 240(10.71) | 464(10.49) |  |  |

BMI, Body mass index; BP, blood pressure; TC, total cholesterol; FPG, fasting plasma glucose; ICH, Ideal cardiovascular health.

**Supplementary Table 3. Model fitting of the association between age at menarche and type 2 diabetes mellitus in rural Chinese women**

| **Models** | **Hosmer and Lemeshow Test** | |
| --- | --- | --- |
|  | **χ2** | ***P*** |
| Model 1 | 61.565 | <0.001 |
| Model 2 | 30.741 | <0.001 |
| Model 3 | 16.616 | 0.034 |
| Model 4 | 10.143 | 0.083 |
| Model 5 | 8.202 | 0.414 |
| Model 6 | 18.228 | 0.020 |
| Model 7 | 17.156 | 0.029 |
| Model 8 | 17.240 | 0.028 |
| Model 9 | 14.897 | 0.061 |
| Model 10 | 13.962 | 0.262 |

Model 1: adjusted for age; Model 2: adjusted as in model 1 plus education level, average monthly individual income, marital status, alcohol drinking; Model 3: adjusted as in model 2 plus family history of diabetes, Age at menopause, parity, the cause of the menopause, use of oral contraceptive pills; Model 4: adjusted as in model 3 plus physical activity; Model 5: adjusted as in model 3 plus body mass index; Model 6: adjusted as in model 3 plus smoking; Model 7: adjusted as in model 3 plus diet; Model 8: adjusted as in model 3 plus blood pressure; Model 9: adjusted as in model 3 plus total cholesterol; Model 10: adjusted as in model 3 plus physical activity, body mass index, smoking, diet, blood pressure, total cholesterol.

**Supplementary Table 4. Sensitivities analyses of the association between age at menarche and type 2 diabetes mellitus according to ideal cardiovascular health metrics further excluding those who reported with cancer and kidney failure (The Henan Rural Cohort Study, collected during 2015–2017, China)**

| **Models** |  | **≤13** | **14** | **15~16** | **17** | **≥18** | **Per 1-year increase** |
| --- | --- | --- | --- | --- | --- | --- | --- |
| **Model 1** |  | 1.200(1.025, 1.406) | 1.088(0.923, 1.282) | Ref. | 0.963(0.828, 1.120) | 0.854(0.753, 0.969) | 0.953(0.932, 0.974) |
| **Model 2** |  | 1.220(1.041, 1.429) | 1.101(0.934, 1.298) | Ref. | 0.953(0.820, 1.109) | 0.841(0.741, 0.954) | 0.948(0.927, 0.970) |
| **Model 3** |  | 1.199(1.020, 1.409) | 1.103(0.933, 1.303) | Ref. | 0.960(0.824, 1.119) | 0.847(0.745, 0.962) | 0.951(0.930, 0.973) |
| **Model 4** |  | 1.206(1.026, 1.418) | 1.101(0.931, 1.302) | Ref. | 0.965(0.828, 1.125) | 0.850(0.748, 0.966) | 0.951(0.930, 0.973) |
| **Model 5** |  | 1.175(0.999, 1.381) | 1.095(0.926, 1.295) | Ref. | 0.983(0.843, 1.146) | 0.891(0.784, 1.013) | 0.962(0.940, 0.984) |
| **Model 6** |  | 1.199(1.020, 1.408) | 1.103(0.933, 1.303) | Ref. | 0.960(0.824, 1.119) | 0.846(0.745, 0.961) | 0.951(0.930, 0.973) |
| **Model 7** |  | 1.216(1.034, 1.430) | 1.116(0.944, 1.320) | Ref. | 0.991(0.850, 1.156) | 0.880(0.774, 1.000) | 0.956(0.935, 0.979) |
| **Model 8** |  | 1.217(1.035, 1.432) | 1.126(0.952, 1.331) | Ref. | 0.964(0.827, 1.124) | 0.861(0.758, 0.979) | 0.951(0.930, 0.973) |
| **Model 9** |  | 1.199(1.020, 1.408) | 1.103(0.933, 1.303) | Ref. | 0.960(0.824, 1.119) | 0.847(0.745, 0.962) | 0.951(0.930, 0.973) |
| **Model 10** |  | 1.215(1.032, 1.430) | 1.127(0.952, 1.334) | Ref. | 1.010(0.865, 1.179) | 0.924(0.812, 1.052) | 0.965(0.942, 0.987) |

Data are odds ratios (95% confidence intervals).

Further excluding those who reported with cancer and kidney failure.

Model 1: adjusted for age; Model 2: adjusted as in model 1 plus education level, average monthly individual income, marital status, alcohol drinking; Model 3: adjusted as in model 2 plus family history of diabetes, Age at menopause, parity, the cause of the menopause, use of oral contraceptive pills; Model 4: adjusted as in model 3 plus physical activity; Model 5: adjusted as in model 3 plus body mass index; Model 6: adjusted as in model 3 plus smoking; Model 7: adjusted as in model 3 plus diet; Model 8: adjusted as in model 3 plus blood pressure; Model 9: adjusted as in model 3 plus total cholesterol; Model 10 adjusted as in model 3 plus physical activity, body mass index, smoking, diet, blood pressure, total cholesterol.
